# “If there’s no stability around them”: experienced therapists’ view on the role of patients’ social world in recovery in bipolar disorder

**DOI:** 10.1186/s13033-017-0166-y

**Published:** 2017-09-20

**Authors:** Marius Veseth, Per-Einar Binder, Signe Hjelen Stige

**Affiliations:** Department of Clinical Psychology, University of Bergen, Christies gate 12, 5012 Bergen, Norway

**Keywords:** Recovery, Social, Relational, Bipolar disorder, Qualitative research, Therapist perspective

## Abstract

**Background:**

Recovery in severe mental illness has traditionally been described as a deeply personal process. At the same time, researchers are increasingly attending to the social nature of such processes. In this article, we aim to supplement the growing knowledge base regarding these social aspects by exploring the perspectives of experienced therapists: how do they view the role of the social world in processes of healing and growth for people with bipolar disorder? And in what ways can the social world impede recovery?

**Methods:**

We conducted 12 semi-structured individual interviews and analyzed the resulting transcripts using a team-based thematic analysis method.

**Results:**

We identified three themes: (a) establishing a sense of belonging; (b) backing ongoing therapy; and (c) relational ripple effects.

**Conclusions:**

We relate our findings to existing theory and research, discuss clinical implications, and highlight study limitations. We argue that our findings underscore the need to integrate an understanding of recovery as a personal and social process in the mental health care services that we provide.

## Background

In the field of mental health, there has been a change in the meaning we attach to the concept of recovery [1, 2]. Traditional descriptions of the term have focused on recovery as an observable clinical outcome rated by professionals in services and research. However, researchers are increasingly understanding recovery as a process that is best understood from the first-person perspective. This shift is characterized by moving from models centering on “recovery from mental illness” to focusing on “recovery in mental illness” [3], from directing treatment and care toward “clinical recovery” to emphasizing the value of “personal recovery” [4]. Capturing the meaning that has emerged from people whom themselves have experienced mental health difficulties, Davidson et al. [5] describe recovery as “a process of restoring a meaningful sense of belonging to one’s community and positive sense of identity apart from one’s condition while rebuilding a life despite or within the limitations imposed by that condition” (p. 25).

As this definition suggests, the active agent in “personal recovery” or “recovery in mental illness,” is the person. It is the individual struggling with his or her mental health who defines goals for the processes of recovery and who needs to do the hard work that this journey entails [3, 4, 6]. This represents an important movement from a more traditional focus on remediation of deficits to discovering the strengths and competencies of people with severe mental illnesses, a shift from paying attention to how we are different to learning what we have in common. At the same time, there may be challenges related to putting the person on center stage. Topor et al. [7] claim, for example, that a recovery perspective risks overlooking important social aspects of recovery processes in developing an individualistic focus. Similarly, Price-Robertson, Obradovic and Morgan [8] call for a new vision of relational recovery that is “based on the idea that human beings are interdependent creatures; that people’s lives and experiences cannot be separated from the social contexts in which they are embedded” (p. 2).

As a result, the research community has recently paid increasing attention to relational factors, demonstrating the value of both interpersonal relationships such as family and friends to recovery processes, as well as the importance of social factors such as housing, occupation and people’s financial situation [9, 10]. In a well-cited systematic evaluation of the literature on recovery processes, Leamy et al. [11] emphasized these social aspects of recovery. One of the main categories in the conceptual framework synthesized in their review is what they term connectedness. This includes the importance of peer support and support groups, relationships, support from others, and being part of the community [11]. As such, connecting to other people is seen as a key process in recovery.

Social aspects are also emphasized in the first-person accounts of people with lived experiences of recovery. In her autobiography, *Madness made me*, Mary O’Hagan [12] portrays this issue in the following way: “The problem with madness is that it maroons us in a place where there is room for only one. Recovery repairs the bridge to belonging” (p. 125). Recovery may accordingly be best understood as both a personal and a social process, as a journey that simultaneously requires individual efforts as well as social re-engagement and community change [7, 9].

For many people struggling with severe mental illnesses, an integral part of recovery processes is the support and treatment offered by mental health services [13, 14]. Therapists’ conceptions of recovery and their perspectives on potential resources in patients’ lives strongly influence the range of choices and actions taken in mental health care. In this article we aim to supplement the growing knowledge base on relational aspects of recovery processes by exploring the perspectives of experienced therapists on recovery in bipolar disorder. This a severe mental disorder characterized by recurrent manic, hypomanic, or depressive episodes. The disorder is estimated to affect between 1 and 3.9% of the population [15, 16] and is associated with significant distress and suffering for the persons affected [17]. As such, it is high-prevalence, high-burden, and for many diagnosed with a bipolar disorder, often long-term.

## Aim

We examine the following research questions: how do experienced therapists view the role of the social world in processes of healing and growth for people with a bipolar disorder? And in what ways can the social world impede recovery?

## Methods

This project was designed as a hermeneutical-phenomenological investigation, with thematic analysis as the practical method of data analysis. The study is phenomenological in its open-minded and experiential focus on how therapists view the role of people’s outside world to processes of recovery [18]. At the same time, we recognize that attempts to describe participants’ experiences will necessarily be based on hermeneutic knowledge that is informed by the researchers’ own preconceptions [19, 20]. Accordingly, our fundamental interpretations of the world will inevitably impact all steps in the process of conducting the research. The main objective of thematic analysis is to establish and describe key categories—themes—that represent important psychological dimensions of participants’ experiences [21].

The article builds on a larger project on recovery in bipolar disorder that examines descriptions of recovery processes from two different perspectives: the views of people with first-hand knowledge of bipolar disorder [22–24] and those of therapists working with people with bipolar disorder [25, 26]. For reflexive purposes, this project was developed in collaboration with a group of service user co-researchers who participated in carrying out the study [27].

### Participants and data collection

The first author led recruitment and collection of data. Purposive and snowball sampling was used to obtain the study sample. First, we identified a few therapists who had broad experience working with persons with bipolar disorders through a regional research network on mood disorders. The initial participants were then asked to help us get into contact with other therapists again. This technique allowed us to target participants with comprehensive experience working with people with bipolar disorder. We consider this highly important because our objective was to gather information-rich participants that would yield insight and understanding of recovery processes in bipolar disorder.

Twelve experienced therapists, seven men and five women, were included in the final sample. Ten were medical doctors specializing in adult psychiatry, and two were clinical psychologists with a specialist license. Their ages ranged from 46 to 68 years at the time of interview, with a mean age of 55. The mean period of practice as therapists was 27 years (range 16–41). All participants were working clinically in the western parts of Norway in the public mental health system, in private practice, or in both.

The participants were interviewed individually based on a semi-structured interview guide. Examples of questions were as follows: what did the patient him- or herself do to promote recovery? What did you do to promote recovery? What did others (family and friends) do to promote recovery? What contributed the most to the positive changes that you observed in this person’s life? What were the biggest barriers for the person in making positive change in his or her life? What does recovery in bipolar disorder mean to you? The mean duration of the interviews was 68 min, with a range of 62–82 min. All interviews were audiotaped and subsequently transcribed verbatim.

### Researchers

All authors are clinical psychologists working at the University of Bergen. At the university, we combine qualitative research with training students in clinical psychology and conducting psychotherapy. The first and third authors are working as associate professors, and the second is a full professor. All authors share an interest in processes of change, growth and healing—both at an individual and a social level, inside as well as outside of treatment and care.

### Data analysis

We used a team-based thematic analysis to describe common, recurrent patterns across our data material [28, 29]. We chose thematic analysis because it lends us the flexibility needed for the exploratory goals of this study, while providing a systematic framework for analysis of the data material. The following six steps developed by Braun and Clarke [17] were used to guide our analytic work:

1. We familiarized ourselves with the data through repeated readings and discussions—both on the phenomena of investigation as well as our own presence in the conduct of the study.
2. We coded the parts of the interviews related to our research questions by identifying units of meaning that represented different aspects of the participants’ experiences. For example, the participants’ descriptions of the importance of relating to others were given the tentative code “having a place in the world outside therapy”.
3. We searched for preliminary themes by examining the codes to identify broader patterns of meaning across the different meaning units.
4. We reviewed and checked the tentative themes, going back and forth between the dataset and our interpretations of the interviews.
5. We defined and named the themes via a process through which we came to an agreement on our understanding of what was important in the data set. The theme “establishing a sense of belonging” was, for example, initially referred to as “creating a sense of belonging.” To communicate that it is, in fact, a real sense of belonging that the participants described their patients achieving here, we ultimately altered this verb.
6. We partnered in writing the article such that the article provides a detailed description of the analytic narratives and data extracts.

## Findings

We organized the participants’ experiences by drawing on the following three themes: (a) establishing a sense of belonging; (b) backing ongoing therapy; and (c) relational ripple effects. These themes are broad descriptions that summarize the experienced therapists’ views of the role of the social world in their patients’ processes of recovery.

### Establishing a sense of belonging

In the interviews, the therapists emphasized the importance for people with bipolar disorder of creating a connection to the social world. We have called this theme “establishing a sense of belonging” to express their descriptions of how community life contributes to healing and growth. Said one of the participants when discussing processes of recovery: “And you need to have some goals in life. Eating pills and spending your days at a psychiatrist’s couch is not an all-time high. So you need to want other things as well.” Another participant described how a solid foundation of social support was particularly crucial for people battling the disabling symptoms of bipolar disorder: “To be able to live with such a severe illness as bipolar, you need that underpinning, you need a firm footing,” the therapist said. Later in the interview, the participant elaborated on this point. As the following quote illustrates, she underscored people’s need for belonging and connection outside the mental health system:

It’s never enough with a therapist and a patient. It’s never enough. No matter how much you try to glorify this with interplay and transference and countertransference. It’s just bullshit. If you haven’t got anything, if you haven’t been loved by anyone but a therapist, it’s no use. You cannot be engaged enough, you never can in a therapist [alone], to the extent that it will make a difference.

Many participants discussed the value of meaningful activities for constructing pathways into community life. One of them emphasized the role of work in a patient’s recovery process in the following way: “He really likes it there, and it’s important because he’s got his identity and his friends and stuff at his workplace.” Although patients’ working lives could be challenging at times, participants generally highlighted work as holding a multitude of positive qualities. These included improving patients’ economic conditions and social status, being important for their feeling of competency and self-worth, and providing them with a necessary structure and social rhythm to their everyday lives. The following quote also illustrates how healing and growth were described as closely related to a patient’s career change: “And people surrounding him at his current workplace […] demonstrate a different… should I say wisdom? A different human knowledge, it seems, and they’re good at inclusion, good at letting him use his resources. I think that’s had an effect.” Another participant described the importance of an active religious life, explaining how a patient had many positive experiences as part of a faith community. The patient needed to avoid some of the more frightening subjects, the therapist said, but found great strength in going to church: “One of the things she says has been vital is her faith. She has eventually developed a very deep trust that good is more powerful than evil.”

In the interviews, some of the therapists warned against the dangers of exclusion for people with bipolar disorder. “The feeling of being parked on the outside of the lives that other people lead, without meaningful human contact. I think that’s very damaging,” shared one of them. According to this participant, mental health and recovery were not so much about symptom relief but instead inextricably linked to processes of finding ones place in the local community: “To me it seems that belonging, meaning in life and some kind of coherence is much more important than if they are in agony or are depressed.” Another participant elaborated further on this aspect. In the interview, he discussed a patient who despite his efforts and advances in handling his symptoms and distress was not viewed as a valued member in the family. “He had more or less become a nonperson to them, and unfortunately that did not break off along with his recovery,” the therapist explained. When a close relative suddenly passed away, this dynamic was consolidated and played out when the patient was not selected as a pallbearer:

He was invited to the funeral, yes, but he was not permitted any of the handles in the casket […] In fact, more distant family members got to escort the casket. And that’s an illustration of this systematic devaluation of him. Accordingly, he became a bystander to this casket being carried out, he wasn’t invited in, and that was really offensive to him. And when all these things came on top of each other, he collapsed and became manic.

### Backing ongoing therapy

Participants discussed several ways that patients’ personal relationships could affect their ongoing therapy and thereby influence processes of recovery. To outline the therapists’ experiences of how the social world could support treatment, we have termed our second theme “backing ongoing therapy.” A central part of this theme was contributing to knowledge and understanding by placing people in proper contexts. Said one of the participants when discussing a patient who did not disclose his everyday life easily: “And when it comes to him, I don’t feel that I have an eyehole into his life.” Despite numerous efforts, the therapist did not manage to include this patient’s family in the ongoing treatment: “I think it would have been reasonable if the wife was here, when he’s so vulnerable to fluctuations. But he doesn’t want that,” the participant explained. Similarly, another participant emphasized the value of collaboration with important others in treatment and care. In the following quote, the therapist describes how home treatment can contribute in building this vantage point for learning more about patients’ everyday life:

Participant: […] And it provides us with a different kind of information. It’s more on the patient’s terms. To come as guests in someone’s home. The roles, the power balance… It’s very different. It often leads to different kinds of collaboration, in my opinion.

Interviewer: And was this useful with this guy?

Participant: Yes, that’s been very, very important. Especially because he’s a guy who would have arrived without a context, as an… Yes, as an individual. ‘Individual’ is Greek and means ‘atom’, and there is something about the atomic in our health system that I don’t like. Things appear fragmented. And they are not. So if you want to see how things are situated, it’s better to be on other arenas than your office or the hospital department. […] And things also get more clear for [patients] when they get to be in their own environment and the wife can provide, what can I call it, affectionate adjustments.

In the interviews, participants also described how personal relationships could contribute more directly to the ongoing clinical work. The therapist above said playfully when discussing this patient’s need to balance his elevated mind: “If you’re bipolar, I think you should to be under your wife’s thumb. That’s the best thing you can do.” Another participant described how carers, in effect, could be co-therapists in the clinical work. When commenting on a patient who had difficulties accepting that she was struggling with her mental health, this therapist noted how she and the patient’s husband were, as she put it, “in the same boat.” When working to help the patient acknowledge her symptoms and distress, the husband and therapist teamed up:

And despite he had only met me once, he had full confidence in me, and… In view of that, he supported me and acted as my extended arm. Because during that week, I’m sure he had to repeat and repeat and repeat […] And I tried to support him on how difficult this was: “No one can do it, except maybe you”. Because you cannot minimize that, on the contrary, you need to give carers full credit.

Here is another excerpt from an interview elaborating how a participant observed family members as important and strong allies in their ongoing therapies. Like the speaker of the quote above, this therapist highlighted how such a role can be a necessity and at the same time quite demanding for the partner:

Participant: If she hadn’t had a husband who was so close, I probably would have to consider coerced admission, because it wouldn’t be safe with regards to suicidality. […]

Interviewer: What does he do to help her?

Participant: They have a trustful relationship and they’re close, they talk together all the time, and… Right now he’s administrating her medication, because it’s not safe for her to keep those pills for herself. I’m thinking of impulses and overdoses. And he collaborates well with me, in the treatment. But there are issues with giving so much responsibility to a spouse. But he feels… In this case it works well and it has worked well in the past. But you need to be attentive as it can get too much or feel unsafe for him.

### Relational ripple effects

In the interviews, participants described how people’s personal relationships could cause symptoms and distress as well as be important keys in their recovery processes. To summarize these experiences, we have called our third theme “relational ripple effects.” Here is a quote illustrating a therapist’s reflections about how the external world also relates closely to what is going on internally: “It is something about… I think that therapy needs to be about gaining stability around them, because then you can help in… If there’s no stability around them, you haven’t got a chance to build stability on the inside.”

Participants described a wide range of pain and suffering brought upon their patients in their personal relationships. These experiences included patients being raped and abandoned by close family members, having carers who denied them access to treatment, experiencing traumatic losses of their loved ones, as well as more everyday problems such as partners who did not provide their patients necessary care and support. One participant also described how fear of losing important others could be a positive motivational force in their ongoing therapy:

He is scared of becoming manic. An anxiety for… He’s got nightmares about making a fool of himself, losing his job, his wife leaving him, his kids loathing him. He’s got a lot of depressive thoughts about becoming manic. And I think that’s been helpful.

Another participant discussed how a well-meaning carer’s efforts to support and help her husband eventually developed into a major barrier for the patient’s processes of recovery. The patient felt he was being choked by his wife’s constant monitoring of him, what he experienced as “a clammy towel around his neck.” The therapist explained:

She was useful in the sense that she gave us the history, she could tell how things were and how they changed. The downside was that he got very sensitive to this. She was walking around observing and monitoring if he was manic or depressed. […] In doing so, everything turns into pathology. I think that was his problem, the wife who really just wanted to help, became a watchdog and took too much care.

Yet another participant discussed a distressing relationship in a patient’s life that was a central challenge to their ongoing therapy. The patient had a mother who also suffered a severe mental illness. In the interview, the therapist described how this situation had impeded their ongoing treatment as the patient was avoiding discussing her own mental health, which again was a barrier for putting the treatment to use in her recovery process:

She has kind of isolated this disorder to her own body. No one should know and no one could help. […] That’s been her strategy, keeping it secret. And she can talk about it now, she has talked a little about it these years I’ve known her: “If I speak about it, everyone will think I’m like my mother. And I won’t. I’m not. I refuse to be” […] And I think that’s been the biggest barrier for her.

As previously mentioned, participants could also perceive important keys to processes of recovery in their patients’ surroundings. Said one of the therapists: “Doing psychoeducation, that’s fascinating […] and perhaps the most valued we’re doing. The families never miss out […] and many understand the idea of seeing things in a different way. And that helps them become empowered and responsible.” Another therapist said: “So the solution to this, as I see it, is relational. It’s not just the patient who needs to change, but also the environment. If not, we will not get anywhere”. In this interview, the participant discussed a patient whose child had been relocated by Child Welfare Services. After a lengthy therapy, he was eventually able to say to his patient: “When you talk about this family reunion and things finally being all right, could it be… Could it be that this is how you want it to be because it is so painful if it is not?” The participant continued:

So that got me thinking that being psychotic, being manic – if the person doesn’t have other things to lean on – is a form of alleviation. It alleviates the suffering. And, sometimes, I think that recovery is about the surroundings understanding that, and accepting that, and making room for that. Accordingly, it is a question of other people providing the space needed [for this], rather than expecting the person itself to relate fundamentally different.

## Discussion

We have presented our analyses of the experiences of the 12 experienced therapists in our study, focusing on the three broad themes identified in their accounts. These findings underscore the multifaceted nature of close relationships and the many paths through which people’s social world can influence their ongoing therapies and recovery processes. The results demonstrate how the therapists viewed patients’ social world as vital, both for recovery processes and for their clinical world. As emphasized in our first theme, to find a place in one’s social community was viewed as being of great importance for recovery. The reason is that community life shapes people’s identity, social roles and contributes to “establishing a sense of belonging.” Our second theme, “backing ongoing therapy,” underscores how patients’ close relationships may also influence treatment and care more directly as the participants described carers as powerful allies and co-therapists. The third theme highlights how the social world may both contribute to and hinder recovery through “relational ripple effects.” This theme emphasizes how people are key to recovery processes and ongoing therapy, and at the same time, it reminds us how not all close relationships are beneficial.

### Belonging and self-agency

The study findings relate to the growing literature on social aspects of recovery. The theme “establishing a sense of belonging,” for example, resonates well with the notion of “connectedness” as a key recovery process, as reported by Tew et al. [9] and Leamy et al. [11]. Furthermore, placing belonging and connection at the heart of the recovery concept may also support the notion of self-agency as central to recovery processes [3, 4]. “To connect is to find roles to play in the world,” claim Jacobson and Greenley [30, p. 483]. As such, belonging and community life is closely related to processes of agency. The concept of self-agency encompasses the belief that the individual him- or herself can impact on their own life [3, 4]. It is the person who is the active agent in shaping recovery processes. Connection, therefore, is—in a sense—about establishing purpose, self-worth and meaning in life. The reason is that these processes have little value without the context of the person’s everyday life and society at large. As Davidson et al. [31] write: “It’s hard to have a sense of belonging to your community without a sense of what you can contribute to in it” (p. 163).

### Implications for practice

An important clinical implication of our findings is the importance of finding helpful ways of integrating people’s social world and their sense of self-agency in their ongoing treatment and care. Several lines of research support this idea. Studies on common factors in psychotherapy demonstrate, for example, that 40% of outcomes in psychotherapy are estimated to be influenced by extratherapeutic factors, such as a patient’s natural social support networks and his or her own resources and efforts [32]. Interestingly, what goes on outside of the therapy room also appears to have affect what occurs on the inside. Studies indicate that positive changes in therapy may be less dependent on the therapeutic alliance in itself when patients have good personal relationships outside therapy [33]. Social support may therefore mediate a major factor regarding the outcome of therapy; the therapeutic relationship. Furthermore, research indicates that the therapeutic relationship is equally important for people with severe mental illnesses [13, 14]. As such, there is clearly a need to find ways of combining our knowledge of recovery as a personal and social process with the mental health services that we provide.

At the same time, as noted in our third theme, some people will carry with them relational experiences that make establishing therapeutic relationships more challenging. In addition, patients’ close relationships may be direct barriers to recovery, rather than facilitators. An important clinical implication of our findings therefore lies in the significance of carefully considering the relational landscapes that make up patients’ social world and exploring the functions these landscapes might have in the person’s processes of recovery. One way of integrating knowledge generated from our findings in treatment and care for people with bipolar disorder is through developing more collaborative practices in mental health care [34]. A diverse team that includes both professionals and natural supporters may, for example, be more likely to be able to think creatively to define and access supports necessary for recovery to occur [35]. A particularly interesting implication may be to implement creative forms of social inclusion through mutual support and peer-organized services [9, 35]. Although not explicitly discussed by the participants in our study, findings indicate that therapists may value and call for such novel partnerships.

### Limitations and strengths

Results are dependent on the participants and the setting in which the research was carried out. The study was conducted within a Norwegian context, thus providing a specific perspective on the phenomenon of focus. Having recruited all participants from the western parts of Norway, and the fact that the majority of them were psychiatrists may decrease the generalizability of the findings. Another limitation of the present study is that therapists will only have partial and incomplete knowledge of their patients’ personal lives. As such, interviews with people with lived experiences and/or their families and friends would likely yield more in-depth information on the role of the social world in recovery processes. At the same time, the perspectives and views of therapists on this issue are clearly important. To create a mental health system that fully encompasses the whole person, we will need to make room for new forms of collaboration that include both people’s professional and personal support network. Such collaboration will require experiential knowledge on the mutual interactions between the person, his or her close relationships, as well as professionals in services. Qualitative studies on recovery has had a center of gravity within research on people with schizophrenia [2–4]. This study adds knowledge to the emerging database on such processes in other clinical populations by exploring those processes for people with bipolar disorder.

## Conclusions

In this qualitative exploration of 12 therapists’ views on the role of their patients’ social world in ongoing treatment and recovery processes, we identified the following broad themes: (a) establishing a sense of belonging; (b) backing ongoing therapy; and (c) relational ripple effects. We argue that our findings underscore the need to integrate an understanding of recovery as a personal and social process in mental health care services.
